# A score prediction model for predicting the heterogeneity symptom trajectories among lung cancer patients during perioperative period: a longitudinal observational study

**DOI:** 10.1080/07853890.2025.2479588

**Published:** 2025-03-20

**Authors:** Yong Yang, Xueqi Tian, Huiling Zhou, Yichao Wang, Yifeng Gu, Ao Qi, Decai Wang, Zhiying Wang, Yabin Gong, Lijing Jiao, Ling Xu

**Affiliations:** a Department of Thoracic Surgery, Shanghai Pulmonary Hospital, Tongji University School of Medicine, Shanghai, China; b Yueyang Hospital of Integrated Traditional Chinese and Western Medicine, Shanghai University of Traditional Chinese Medicine, Shanghai, China

**Keywords:** Lung cancer, prediction model, quality of life, surgery, symptom burden, symptom trajectory

## Abstract

**Introduction:**

Patients undergoing video-assisted thoracoscopic surgery (VATs) for lung cancer (LC) frequently experience prolonged symptoms that can significantly affect their quality of life (QoL).

**Patients and methods:**

This study employed a longitudinal observational design. The MDASI and QLQ-C30 were utilized to evaluate symptoms and QoL one day before surgery, as well as at 1 day, 2 weeks, and 1, 2, and 3 months post-surgery. Latent class growth modeling (LCGM) was employed to identify heterogeneous trajectories. By Logistic regression analysis, a score prediction model was developed based on predictive factors, which was internally validated utilizing 1000 bootstrap samples. The SHaply Additive Explanations (SHAP) was used to calculating the contribution of each factor.

**Results:**

205 participants participated in this study. The predominant postoperative complaints included fatigue, shortness of breath, pain, and coughing. Two distinct classes of symptom trajectories were identified: ‘severe group’ and ‘mild group’. Four independent predictors of heterogeneous symptom trajectories were used to develop a scoring model. The area under the receiver operating characteristic curve for this model was 0.742 (95% CI: 0.651–0.832). And the calibration curves demonstrated strong concordance between anticipated probability and actual data (mean absolute error: 0.033). Furthermore, the decision curve analysis (DCA) indicated higher net benefit than other four single factors. SHAP highlighted WBC and surgical duration time as the most influential features.

**Conclusions:**

We established a score model to predict the occurrence of severe symptom trajectories 3 months postoperatively, promoting recovery by advancing rehabilitation plan based on preoperative and surgical situation.

**Registration:**

ClinicalTrials.gov (ChiCTR2100044776).

## 1. Introduction

The latest cancer data from 2024 show that lung cancer (LC) is the most numerous and deadly type of malignant tumor [1]. Currently, surgery remains the most efficacious treatment for patients with stage I–IIIA non-small cell lung cancer (NSCLC) patients [2]. Compared to traditional open surgery, video-assisted thoracoscopic surgery (VATS) results in less trauma, intraoperative bleeding, complications, and impacts on cardiopulmonary function, as well as faster postoperative recovery for patients [3,4]. However, there are still unavoidable side effects, such as chronic cough, pain, and fatigue, which may compromise quality of life (QoL) [5,6].

Therefore, it is imperative to anticipate and recognize severe symptoms promptly and actively prevent their occurrence to promote rapid recovery for patients. However, most current research on symptoms focuses on the main symptoms and progression trajectory of symptoms after surgery, with few studies exploring predictors of symptoms and constructing predictive models [7–9]. One study identified heterogeneous symptom trajectories among LC patients in the first year after surgery and identified factors influencing severe symptoms but did not construct a predictive model [10].

Previous research has indicated that the incidence and severity of symptoms in LC patients change over time within the first 3 months after surgery, with all symptoms improving to preoperative levels by the end of the third month [11]. This highlights the need for further attention to heterogeneous symptom development trajectories in the first 3 months after surgery and to identify key populations in need of rehabilitation as early as possible.

This study aimed to delineate the symptom burden of LC patients 3 months post-surgery, identify the trajectory of symptoms, thoroughly examine predictors of this trajectory, and develop a clinically applicable scoring model for predicting the onset of severe symptoms.

## 2. Methods

### 2.1. Research design and participants

The convenience sampling method was employed to select LC patients who received surgical therapy at two tertiary hospitals from February to November 2023 in China. The project sought to gather and analyze preoperative prognostic factors. A longitudinal evaluation of symptom burden was performed 3 months post-surgery in patients with LC who had been included in the study before the procedure. The follow-up period extended from February 2023 to February 2024. Patients underwent routine postoperative care in accordance with established recommendations and consensus, and received proactive symptom management only if they actively requested medical assistance. In the event of serious symptoms, individuals may pursue online consultations or visit nearby hospitals. This research was conducted in accordance with the Strengthening the Reporting of Observational Studies in Epidemiology (STROBE) guidelines.

The inclusion criteria were as follows: patients who had undergone single-port VATs for LC, diagnosed by paraffin pathology, aged 18 to 80 years, and possessed the capacity to comprehend and consent to sign the informed consent form.

The exclusion criteria included patients who underwent preoperative radiation or chemotherapy; individuals with uncontrolled neurological, urinary, digestive, endocrine, or other systemic problems; and those with mental or cognitive impairments.

Data on predictive variables were gathered by trained investigators one day prior to surgery (T0), while information regarding symptom burden and QoL was obtained through face-to-face interactions one day before and after surgery (T1). After discharge, trained investigators follow up patients *via* telephone on 2 weeks (T2), 1 month (T3), 2 months (T4), and 3 months (T5) post VATs. Electronic questionnaires were send to patients *via* text messages and collected on the day of follow-up. A questionnaire with a response rate of more than 95% was qualified.

### 2.2. Sample size

A study showed that the prevalence of severe symptoms in LC is approximately 24.53%, with a 95% confidence interval and a margin of error of 0.13 [10,12]. The PASS 26 determined the sample size to be 182. Considering a 10% loss rate of follow-up, the final sample size was established to be a minimum of 202 cases.

### 2.3. Ethic

All participants provided informed consent prior to participating in the study. The study adhered to the principles of the ‘Declaration of Helsinki’. This study was approved by the Ethics Committee of Yueyang Hospital of Integrated Traditional Chinese and Western Medicine affiliated with Shanghai University of Traditional Chinese Medicine (Number: 2020-038).

### 2.4. Measurements

#### 2.4.1. Symptom burden and symptom trajectories

Symptom burden is defined as the severity and incidence of symptoms and their impact on the patient’s daily activities, reflecting the significant distress suffered by the patient as a result of one or more symptoms [13–15]. The MD Anderson Symptom Inventory (MDASI) is a self-assessment questionnaire that contains comprehensive symptoms. It was created by the MD Anderson Cancer Center at the University of Texas in 2000. This scale is widely used in various pathological types of cancer and in various treatment modalities [16,17]. Professor Pingping Li of the Beijing Institute of Cancer Prevention and Treatment added specific symptom items of traditional Chinese medicine (TCM) to the 13 fundamental symptom items of the MDASI, creating the TCM segment of the MDASI scale (MDASI-TCM) [18]. There is no MDASI version for postoperative lung cancer patients. In this study, 11 symptom items including cough, phlegm, palpitation, sweating, bitter mouth, oral ulcer, diarrhea, constipation, irritability, hot hands and feet, and abdominal distension were added to the core symptom items of MDASI, which formed MDASI-TCM. Patients reported their symptoms using the MDASI-TCM by themselves, which comprised 24 symptom items. The scale ranged from 0 to 10, with 0 indicating ‘absent’ and 10 representing ‘extremely severe’. Severe symptoms were defined as symptom score ≥7. In this study, the Cronbach’s α coefficient of the MDASI-TCM scale was 0.945, and the Cronbach’s α coefficient of each dimension ranged from 0.940 to 0.946.

The composite score of the top ten symptoms in terms of incidence was used to assess symptom trajectories, which consisted of tiredness, shortness of breath, pain, sleep disturbance, cough, dry mouth, anxiety, phlegm, sweating, and forgetfulness. Symptom improvement was defined as a decrease of ≥10 points in the total score from the T1 to the first occurrence, and a decrease of ≥10 points from T1 for two consecutive times. Symptom improvement time was defined as the time from day 1 of surgery (T1) to the first occurrence of score reduction of ≥10 points, and two consecutive ≥10 points reduction from T1 [19].

#### 2.4.2. QoL

The Quality of Life Questionnaire-Core 30 (QLQ-C30), designed by the European Organization for Research and Treatment of Cancer (EORTC), was used to assess QoL [20]. The scale contains 30 items categorized into five functional domains, one general health domain, and nine symptom domains. This study revealed the functions of the body, role, social function, cognition, and emotion. An elevated score signifies an improved QoL.

#### 2.4.3. Predictive variables

##### 2.4.3.1. Physiological and biochemical parameters

Information pertaining to age, sex, body mass index (BMI), and past medical history was collected by inquiries and examinations. The white blood cell (WBC) (g/L), neutrophils (NEUT) (g/L), total protein (TP) (g/L), hemoglobin (Hb) (g/L), lactate dehydrogenase (LDH) (U/L), platelets (PLT) (g/L), heparin-binding protein (ng/L), and creatinine (µmol/L) were derived from the blood biochemical examination of the patients. This investigation identified comorbidities, such as hypertension, diabetes, pulmonary tuberculosis, asthma, pulmonary bullae, chronic bronchopneumonia, various lung disorders, and a personal history of malignant tumors.

##### 2.4.3.2. Social and economic status

This encompasses educational attainment, smoking history, and occupational classifications.

##### 2.4.3.3. Constitution questionnaire

The TCM constitution comprises a balanced and biased constitution. This is assessed by the ‘Classification and Judgment of Traditional Chinese Medicine Physique’ scale, which grades of each item from 0 to 5 for each item [21].

##### 2.4.3.4. *P*ulmonary *function*

Forced vital capacity (FVC) (%) and forced expiratory volume in 1 s (FEV1) (%) were obtained from the pulmonary function assessment report.

##### 2.4.3.5. Surgical information

The resection scope, surgical site, surgical time, intraoperative blood loss, pathological type, and pathological stage were obtained from surgical records and pathological reports.

##### 2.4.3.6. QoL

The five functional domains of QLQ-C30 at T0 were used to represent the patient’s QoL at baseline.

### 2.5. Statistical analysis

The mean (±standard deviation) and frequency (ratio) were used for statistical description. The χ^2^ test was employed for categorical data, while the Mann–Whitney *U* test was used for continuous data. The Log rank test was used to compare the symptom improvement time between groups. Statistical significance was set at *p* < 0.05.

The cumulative score of the ten most prominent symptoms within 3 months post-operation was used to ascertain diverse symptom trajectories. The Mplus 8.7 was employed to discern patient clusters exhibiting varying symptom trajectories through latent class growth modeling (LCGM) and growth mixture modeling (GMM). These methods were selected for their ability to accommodate unequally spaced or absent observations and incorporate patients with sporadic missing data. A linear mixed effects model was used to fit the heterogeneous trajectories. Since patients’ symptoms showed a trend of first aggravation and then remission from T0 to T5, a segmented heterogeneity trajectory curve model was established (taking the first day after surgery as the inflection point). The adequacy of symptom trajectories was assessed using the Akaike information criterion (AIC), Bayesian information criterion (BIC), adjusted Bayesian information criterion (aBIC), entropy, bootstrapped likelihood ratio test (BLRT), and Lo–Mendell–Rubin (LMR). The smaller the statistical values of the fitting index AIC, BIC and aBIC, the better the model fitting is. The higher the entropy value, the higher the classification accuracy. The statistical values of LRT, LMR and BLRT were *p* < 0.05, which meant that the model fitting degree of K categories was better than K-1 categories. A two-class mixed model was employed to measure the extent of symptom change over time for each patient cluster using postoperative day 1 as the inflection point.

Univariate logistic regression analysis was used to examine the predictive determinants of symptom trajectories. Variables exhibiting a *p* value below 0.1 in the univariate study were incorporated into a multivariate logistic regression analysis (method: Forward, LR. *p* < 0.05). A scoring model was developed based on the outcomes of multivariate logistic regression.

The efficacy of the scoring model was assessed using discriminating and calibration metrics. The area under the receiver operating characteristic (ROC) curve (AUC) was used to evaluate the model’s discrimination, with a value over 0.70, which is indicative of good discriminatory performance. A visual calibration map was employed to compare the anticipated and actual probabilities of varied symptom trajectories and assess the calibration of the model. The model underwent 1000 bootstrap resamples for internal validation to evaluate its predictive accuracy. The net advantages of a specific threshold probability were assessed using decision curve analysis (DCA) to determine the therapeutic utility of the scoring model. The model results were interpreted by calculating the contribution of each feature to the predicted results by SHaply Additive Explanations (SHAP).

The statistical analysis and graphics were performed utilizing SPSS version 26.0 (IBM SPSS Statistics, Armonk, NY, USA) and R version 24.04.2 (R Foundation for Statistical Computing, Vienna, Austria), employing the ‘rms’, ‘pROC’, ‘rmda’ and ‘DALEXtra’ statistical packages.

## 3. Results

### 3.1. Participant characteristics

After screening 260 patients, 205 were enrolled (Supplemental Figure 1). The predominant demographic of the participants was female, at 60.00% of the total, 90 participants (43.90%) were over the age of 60, 78 participants (30.05%) were retired, and 43 (20.98%) had a primary education or less. Further details are presented in Table 1.

**Figure 1.**
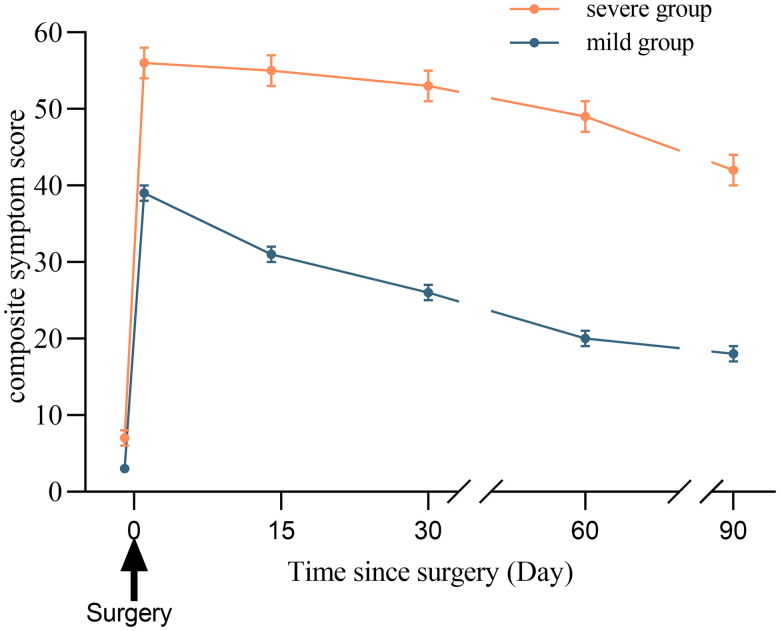
The total composite symptom scores of the two groups.

**Table 1.** Fundamental demographic and clinical attributes of participants (*n* = 205).

Variables	*n* (%)	
Sex		
Male	82	(40.00)
Female	123	(60.00)
Age (years)		
≤60	115	(56.10)
>60	90	(43.90)
BMI (kg/m^2^)		
Below 18.5	6	(2.93)
18.5–23.9	81	(39.51)
24.0–27.9	96	(46.83)
28 and above	22	(10.73)
Occupation		
Leaders of state organs, enterprises and institutions	8	(3.90)
Office staff	9	(4.39)
Professional technician	15	(7.32)
Production and transportation staff	10	(4.88)
Farming, forestry, livestock industry, and fishery producers	14	(6.83)
Social manufacturing and life services employees	13	(6.34)
Retiree	78	(38.05)
Liberal professions	38	(18.53)
Others	20	(9.76)
Education		
Primary school and below	43	(20.98)
Junior middle school	69	(33.66)
Senior middle school	49	(23.90)
Undergraduate	40	(19.51)
Graduate students	4	(1.95)
Constitution		
Balanced constitution	53	(25.85)
Biased constitution	152	(74.15)
Lung disease		
No	170	(82.93)
Yes	35	(17.07)
Hypertension		
No	132	(64.39)
Yes	73	(35.61)
Diabetes		
No	179	(87.32)
Yes	26	(12.68)
Smoking		
No	131	(63.90)
Yes	74	(36.10)
Personal history of malignant neoplasm		
No	187	(91.22)
Yes	18	(8.78)
Tumor location		
Left superior lobe	51	(24.88)
Left inferior lobe	35	(17.07)
Right superior lobe	59	(28.78)
Right middle lobe	17	(8.29)
Right inferior lobe	26	(12.69)
More than one location	17	(8.29)
Extent of surgery		
Leaf cutting	66	(32.20)
Segmented cutting	68	(33.17)
Wedge-shaped cutting	71	(34.63)
Surgical duration time (h)		
<2	139	(67.80)
≥2	66	(32.20)
Intraoperative blood loss (ml)		
≤50	92	(44.88)
>50	113	(55.12)
Pathological type of lung cancer		
Adenocarcinoma *in situ*	51	(24.88)
Microinvasive adenocarcinoma	53	(25.85)
Invasive adenocarcinoma	79	(38.54)
Others	22	(10.73)
Postoperative adjuvant therapy		
Chemotherapy	14	(6.83)
Immunotherapy	2	(0.98)
Targeted therapy	4	(1.95)
None	185	(90.24)
pTNM stage (8th edition)		
0	51	(24.88)
IA1	40	(19.51)
IA2	54	(26.34)
IA3	28	(13.66)
IB	11	(5.37)
IIA–IIB	9	(4.39)
IIIA	12	(5.85)

*Note*. BMI, body mass index; pTNM, pathologic tumor-node-metastasis staging system; SD, standard deviation.

### 3.2. Symptom burden

The most severe symptoms reported by patients one day post-surgery were pain (6.64 ± 0.12), fatigue (6.27 ± 0.13), disturbed sleep (4.94 ± 0.16), shortness of breath (4.30 ± 0.15), and dry mouth (4.28 ± 0.16). The ten most common postoperative symptoms were fatigue (3 months average: 91.75%), shortness of breath (90.80%), pain (88.61%), coughing (84.08%), disturbed sleep (77.35%), dry mouth (74.25%), distress (72.37%), expectoration (71.50%), sweating (64.82%), and remembering (60.74%). More details are provided in Supplementary Tables 1 and 2.

### 3.3. Symptom trajectories

Among the results of the GMM and the LCGM, the statistical values of LMR and BLRT were *p* < 0.05 in ‘2C’ and ‘3C’ of LCGM, indicating a significantly better fit. On the other hand, the entropy representing the classification accuracy of the model is larger in ‘2C’ than ‘3C’ of LCGM. Therefore, a two-class trajectory model was developed using the LCGM, focusing on the 10 most common symptoms (Supplementary Table 3). Class 1, including 80.49% of patients (165/205), was designated the ‘mild group’ owing to their low baseline scores and a moderately declining trajectory until one day post-surgery, followed by a rather swift decreasing trend until 3 months post-surgery. Class 2, including 19.51% of the patients (40/205), exhibited elevated initial symptom scores and a sharply ascending trajectory until one day post-surgery, followed by a mild decline until 3 months post-surgery. This group’s overall trajectory surpassed that of the ‘mild group’ at six-time intervals, resulting in its designation as the ‘severe group’. Additional information is provided in Figure 1.

### 3.4. Differences between the ‘mild group’ and ‘severe group’ in symptom burden and QoL

**Figure 2.**
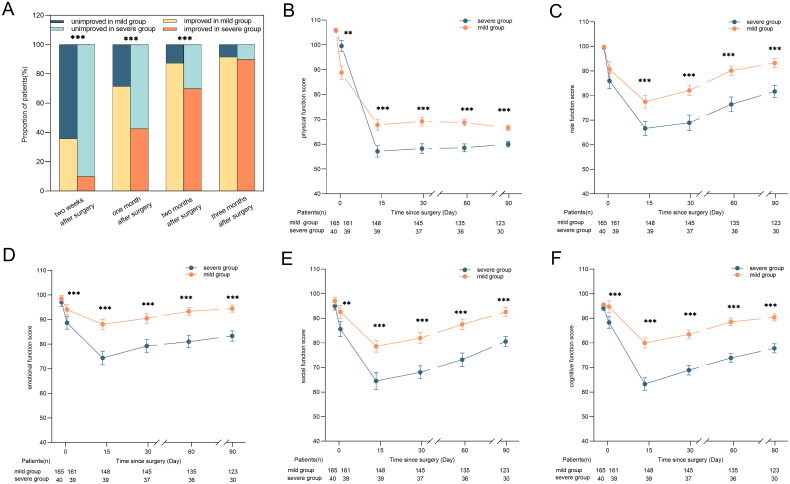
(A) Proportions of patients exhibiting better and unimproved composite symptom scores on the MDASI-TCM. (B) Physical function scores on the QLQ-C30 from enrollment to 3 months. (C) Role function scores in the QLQ-C30 from enrollment to 3 months. (D) The scores of emotional function from enrollment to 3 months. (E) The scores of social function in QLQ-C30 from enrollment to 3 months. (F) Cognitive function scores in the QLQ-C30 from enrollment to 3 months. QLQ-C30, European Organization for the Research and Treatment of Cancer Quality of Life Questionnaire Core 30. MDASI-TCM, MD Anderson Symptom Inventory-Traditional Chinese Medicine; **p < 0.05, ***p < 0.001.

The log-rank test indicated that the ‘mild group’ exhibited symptom improvement sooner (1-month post-surgery) than the ‘severe group’ (2 months post-surgery) (HR = 1.865, 95% CI: 1.197–2.907, *p* = 0.006). Furthermore, in addition to enhanced role performance, the ‘mild group’ also demonstrated better the function of body, role, cognition, and emotion from 1 day to 3 months post-surgery compared to the ‘severe group’ (all *p* < 0.05). The results are shown in Figure 2A–F.

### 3.5. Analysis of predicting factors for varied symptom trajectories using univariate and multivariate methods

There were 29 cases (14.15%) with missing LDH data and 57 cases (27.80%) with missing heparin-binding protein data. The mean interpolation method was used to fill the missing data. According to the Jordan index, the truncation values of the continuity detection variables PLT, NEUT, WBC, Hb, TP, AST, creatinine, LDH, heparin-binding protein, FVC, and FEV1 were determined as follows: 190.5 × 10^9^/L, 5.65 × 10^9^/L, 7.76 × 10^9^/L, 129.5 g/L, 69.85 µmol/L, 15.25 U/L, 53.35 µmol/L, 170.9 U/L, 50.67 ng/L, 108.15%, 99.65%. Supplementary Table 4 illustrates that the univariate regression analysis identified 11 factors associated with symptom trajectories. These included history of diabetes (OR = 2.528, 95% CI = 0.032–6.191, *p* = 0.043), constitution (OR = 2.872, 95% CI = 1.061–7.771, *p* = 0.038), social function (OR = 0.952, 95% CI = 0.897–1.009, *p* = 0.099), surgical duration (OR = 3.361, 95% CI = 1.649–6.851, *p* = 0.001), intraoperative blood loss (OR = 2.196, 95% CI = 1.046–4.612, *p* = 0.038), PLT (OR = 2.602, 95% CI = 0.870–7.783, *p* = 0.087), NEUT (OR = 2.970, 95% CI = 1.071–8.231, *p* = 0.036), WBC (OR = 2.704, 95% CI = 1.095–6.671, *p* = 0.031), TP (OR = 1.991, 95% CI = 0.932–4.255, *p* = 0.075), LDH (OR = 2.316, 95% CI = 1.149–4.667, *p* = 0.019), and heparin binding protein (OR = 4.931, 95% CI = 1.133–21.471, *p* = 0.034).

Considering the sample size of 205 in this study, it is appropriate to include 11 variables in the multivariate regression analysis according to the rule of 10-fold events per variable [22]. In the multivariate logistic regression model, constitution (OR = 3.832, 95% CI = 1.323–11.099, *p* = 0.013), surgical duration (OR = 3.053, 95% CI = 1.441–6.466, *p* = 0.004), LDH level (OR = 2.190, 95% CI = 1.020–4.703, *p* = 0.044), and WBC count (OR = 2.748, 95% CI = 1.022–7.391, *p* = 0.045) were independent predictors of heterogeneous symptom trajectories. Additional information is provided in Table 2.

**Table 2.** Multivariable logistic analysis and assigned scores for predictors of heterogeneous symptom trajectories among LC patients during the perioperative period (*n* = 205).

Variables	*B*	SE	χ^2^	*p* value	OR	95% CI	Score
Constitution							
Balanced constitution					1.000		0
Biased constitution	1.343	0.543	6.130	0.013	3.832	1.323–11.099	4
Surgical duration time (h)							
<2					1.000		0
≥2	1.116	0.383	8.496	0.004	3.053	1.441–6.466	3
WBC (×10^9^/L)							
<7.76					1.000		0
≥7.76	1.011	0.505	4.009	0.045	2.748	1.022–7.391	3
LDH (U/L)							
<170.9					1.000		0
≥170.9	0.784	0.390	4.039	0.044	2.190	1.020–4.703	2

LDH, lactate dehydrogenase; WBC, white blood cell; OR, odds ratio.

### 3.6. Development and performance assessment of the score prediction model

Based on Table 2, a score model that adopted four independent predictors was developed. The β values of each risk factor were rounded off to integers and assigned to their respective factors, as shown in Table 2. Patients were classified by calculating the total score of the four variables: constitution (balanced = 0, biased = +4), surgical duration (<2 *h* = 0, ≥2 *h* = +3), WBC count (<7.76 × 10^9^/L = 0, ≥7.76 × 10^9^/L = +3), and LDH level (<170.9 U/L = 0, ≥170.9 U/L = +2). The optimal critical value of the total score was calculated to be 4.5, and the rounding was 5 points. Therefore, those who scored ≥5 points in the scoring model were classified into the severe group and those who scored <5 points were classified into the mild group.

**Figure 3.**
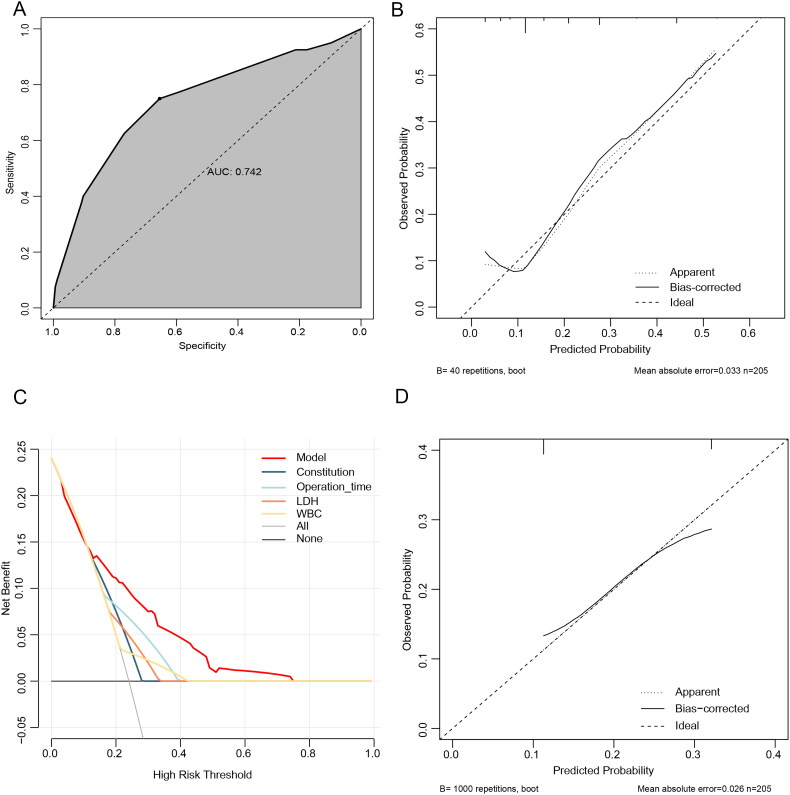
(A) ROC curve of the model (B). Model calibration curves. The *x*- and *y*-axes depict the expected and observed occurrence of heterogeneous symptom trajectories, respectively. (C). DCA curves of the model. The black line indicates that no cases were in the severe group, while the gray line indicates the opposite. (D). Calibration curves for 1000 bootstrap internal validations of the model. DCA, decision curve analysis. AUC, area under the receiver-operating characteristic curve; ROC, receiver-operating characteristic.

The model demonstrated strong discrimination (AUC: 0.742, 95% CI: 0.651–0.832, Figure 3A) with a specificity of 0.806, while the correction curve indicated that the expected probability of heterogeneous symptom trajectories derived from the scoring model closely matched the actual probability (mean absolute error (MAE): 0.033, Figure 3B). In this study, out of 205 LC patients, 40 were classified into the severe group and 26 (32.6%) developed severe disease. In the mild group, there were 165 cases, and 55 cases (13.9%) actually developed severe. Therefore, the positive predictive value of the risk prediction score model was 65.0%, negative predictive value was 66.67%, and overall accuracy was 66.34%. Furthermore, the DCA curve showed that the net benefit was higher than those of the other four factors (Figure 3C). And when the threshold probability is about 0.12–0.75, the net benefit of using the model to predict ‘severe group’ was higher, and the wide threshold probability indicated that the model was of value in clinical. When verified, the AUC was 0.658 (95% CI: 0.575–0.741), and the Hosmer–Lemeshow test yielded an MAE of 0.026 (Figure 3D).

**Figure 4.**
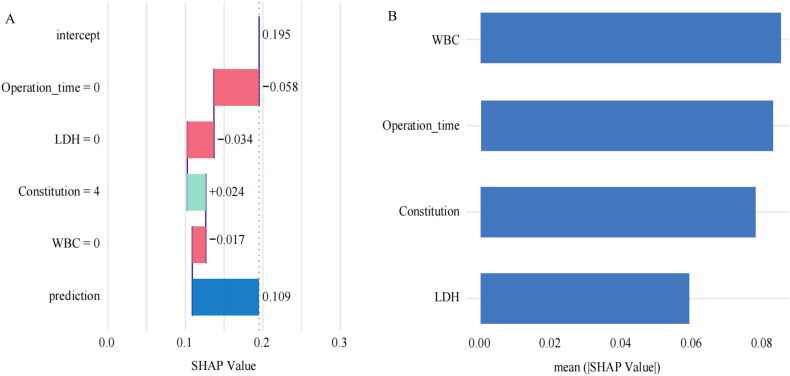
(A) Attributes of characteristics in SHAP. Each line represents a feature, and the abscissa is the SHAP value. (B). Feature importance ranking as indicated by SHAP. The matrix diagram describes the importance of each covariate in the development of the final prediction model. SHAP, SHaply Additive Explanations.

We used SHAP to illustrate how the chosen variables predict the model’s creation. Our model’s four features were displayed in Figure 4A. Patients undergoing lung cancer surgery were more likely to experience severe symptoms if they had elevated WBC, LDH, bias constitution, and a longer operation length. Figure 4B showed the ranking of the four risk factors assessed by the average absolute SHAP value, where the *X* axis SHAP value indicated the importance of the predictive model. Operation time and WBC were especially crucial.

## 4. Discussion

Patients undergoing VATs for LC frequently endure severe symptoms within the first 3 months after surgery, which can significantly impact their QoL. Therefore, we conducted an experiment to ascertain the progression of symptoms at 3 months post-surgery, identify symptom trajectories, and explore pertinent variables to create a predictive scoring model. Ultimately, we identified two symptom trajectories in LC patients during the 3 months after surgery: the ‘severe group’ and the ‘mild group’. Additionally, this study determined the factors that contribute to these heterogeneous symptom trajectories, including constitution, surgical duration, WBC count, and LDH. Based on these findings, we developed a scoring model to predict individuals in the potentially ‘severe group’.

First, we observed a consistent decline in symptom scores within 3 months after surgery in patients with LC. The most frequent symptoms reported were pain, fatigue, shortness of breath, and disturbed sleep, which aligns with prior research [23,24]. Even though there have been a lot of research on symptom burden recently, the most of them just address one symptom or the factors that influence it. They also don’t create predictive models or fully take into account a number of typical postoperative symptoms of lung cancer. 264 patients with varying pain trajectories following lung cancer surgery were identified by Gjeilo KH using a potential class mix model (LCMM). These subgroups included patients with higher comorbidity scores and concurrent use of pain and psychotropic medications prior to surgery as factors for pain trajectories [25]. Three distinct fatigue tracks of 202 lung cancer patients who underwent surgery and adjuvant chemotherapy were identified by LCGM analysis: a persistently high fatigue group, a growing fatigue group, and a no fatigue group [26]. Cancer-related fatigue tracks were associated with depressive personality type, high mental resilience, and cancer stage. Based on the overall symptom score, our study categorized the ten most prevalent symptoms following lung cancer surgery into two symptom trajectory subgroups: the ‘mild group’ (80.49%) and the ‘severe group’ (19.51%). According to our symptom trajectory profile, some people may be able to improve their symptoms on their own without the need for any medical procedures. As such, it is critical to determine which individuals actually need care. This implies that in order to properly manage the symptoms of various classes of LC patients, customized interventions are required. In order to deliver individualized preventive interventions, it is imperative to comprehend the traits of these various groups and recognize them early.

The clusters of trajectories in our study suggest that certain factors, such as biased constitution, longer surgical duration, higher WBC count, and higher LDH levels, increase the probability of a more severe symptom burden post-surgery. Patients with a longer surgical duration are more likely to experience heterogeneous symptom trajectories. This may result from a combination of circumstances, including lobar resection, intercostal nerve damage, and pleural irritation caused by the chest tubes. Intraoperative factors, such as single-lung ventilation and traumatic stress, can result in the release of oxygen free radicals and inflammatory cytokines, resulting in local and systemic lung inflammation and increasing the risk of postoperative complications. Longer operation times are associated with a higher risk for these factors, which can affect the speed of patient recovery [27–29]. LDH is a peripheral blood immune indicator that has been linked to pro-inflammatory conditions and their impact on the immune response to cancer [30]. It is also a significant predictor in several prognostic tools for NSCLC, including the Pulmonary Immune Prognostic Index (LIPI) [31], EPSILoN score [32], and Gustave Roussy immune score [33]. These tools have been proven to be effective in predicting outcomes for NSCLC patients. LDH is crucial for the transformation of cancer cells into Warburg metabolism, promoting sustained growth and division. Elevated LDH levels reflect high tumor burden and inflammatory reactions [34,35]. This is usually caused by a ‘driver mutation’ that activates an effector of the growth factor pathway or inactivates a suppressor. For example, in NSCLC, KRAS mutations promote the cell cycle from the G1 phase to the S phase [36]. Increased LDH is not only significantly correlated with poor prognosis in NSCLC patients undergoing targeted therapy or surgery [37,38], but is also associated with poor postoperative recovery. Oxalates (LDH-A inhibitors) significantly inhibited the proliferation of NSCLC cells and were much less toxic in normal cells. These results suggest the potential use of targeting LDH-A in the treatment of NSCLC [39]. Additionally, we found that a higher WBC count was a risk factor for heterogeneous symptom trajectories. A previous study corroborated these findings [40]. Overproduction of granulocyte colony-stimulating factor (G-CSF), granulocyte macrophage colony-stimulating factor (GM-CSF), and interleukin-6 in lung cancer has been linked to leukocytosis [41]. Neutrophils, the most abundant peripheral white blood cells, have also been identified as a prognostic factor for tumors [42]. The study of TCM constitution examines the overall body condition, which is influenced by both innate and acquired factors [43]. And the probability of encountering severe symptoms in the postoperative phase is heightened in individuals with a biased constitution. The constitution mirrors the attributes of the human body, and physical predispositions can influence disease outcomes and slow the progress of down rehabilitation progress [44].

Therefore, it is crucial to identify patients at risk of severe postoperative symptoms through preoperative assessments and to minimize their operative time to mitigate potential risks. Surgery can trigger systemic inflammation *via* proinflammatory and compensatory anti-inflammatory responses; however, this can be alleviated through minimally invasive techniques and autonomous single lung ventilation [45]. Such approaches have been shown to enhance patients’ QoL and overall health outcomes [46]. Current clinical practices emphasize early mobilization, early walking, breathing exercises, and expectoration during hospitalization, which have been demonstrated to reduce the expression of proinflammatory cytokines. Furthermore, targeted rehabilitation strategies, including aerobic and resistance training, inspiratory muscle training, and postoperative breathing exercises [47], have proven effective in accelerating recovery. Traditional Chinese exercises, such as Liuzijue and the use of Chinese herbal medicine, have also been shown to alleviate physiological symptoms and improve QoL in patients recovering from early-stage lung cancer surgery [48]. Additionally, optimizing nutrition, improving sleep quality [49], and implementing fatigue–relief measures can further reduce inflammation and support postoperative rehabilitation [50]. For patients predisposed to inflammation, it is essential to enhance their physical condition through lifestyle modifications, regular exercise, a balanced diet, and, when necessary, pharmacological interventions during the recovery phase [51]. This multifaceted approach ensures comprehensive care and promotes optimal recovery outcomes. Patients in the ‘severe group’ of LC surgery should receive at least 3 months of postoperative rehabilitation guidance to facilitate their recovery process.

We created a predictive model utilizing multivariate regression analysis with four indicators, capable of accurately forecasting the likelihood of diverse symptom trajectories in LC patients during the 3-month postoperative period. Furthermore, we developed a scoring model to improve the clinical use and practicality of the prediction model, facilitating the rapid assessment of a patient’s probability of encountering diverse severe symptom trajectories. The model’s performance was assessed using ROC, calibration, and DCA curves, all of which demonstrated strong discrimination, calibration, and therapeutic benefits.

Nonetheless, there are still some limitations to this study. First, it is important to note that all data related to symptoms were self-reported by the patients. Although our researchers exercise strict quality control, the possibility of either overestimation or underestimation of the actual metrics is hard to avoid. To improve the accuracy of the data, future studies could consider using objective measures or incorporating data from medical records. Additionally, the statistics on emergency department visits and long-term QoL were not available for this study. As a result, the prediction model can only forecast the trajectory of symptoms within 3 months after surgery. The long-term trajectory of symptoms needs further long-term follow-up. Furthermore, the rehabilitation suggestions provided in this study were based on previous research by other researchers. While these suggestions may be helpful, more detailed and specific rehabilitation programs should be developed and studied in the future. Finally, owing to the limited number of patient collection centers and the relatively small sample size, this study was restricted to internal validation and did not incorporate external validation. This limitation may affect the generalizability and applicability of the results. To enhance the reliability and robustness of the findings, future research should aim to include a larger and more diverse patient cohort, encompassing multiple regions and varying levels of medical care.

## 5. Conclusion

Our study established a score model to predict the occurrence of heterogeneous severe symptom trajectories 3 months postoperatively and provided evidence for identifying patients in need of more effective symptom management to enhance functional rehabilitation.

## Supplementary Material

Supplemental Material
