# Supplementary figures and images for: A score prediction model for predicting the heterogeneity symptom trajectories among lung cancer patients during perioperative period: a longitudinal observational study
Source: Ann Med. 2025 Mar 20;57(1):2479588. doi: 10.1080/07853890.2025.2479588 (PMC11934189; doi:10.1080/07853890.2025.2479588)

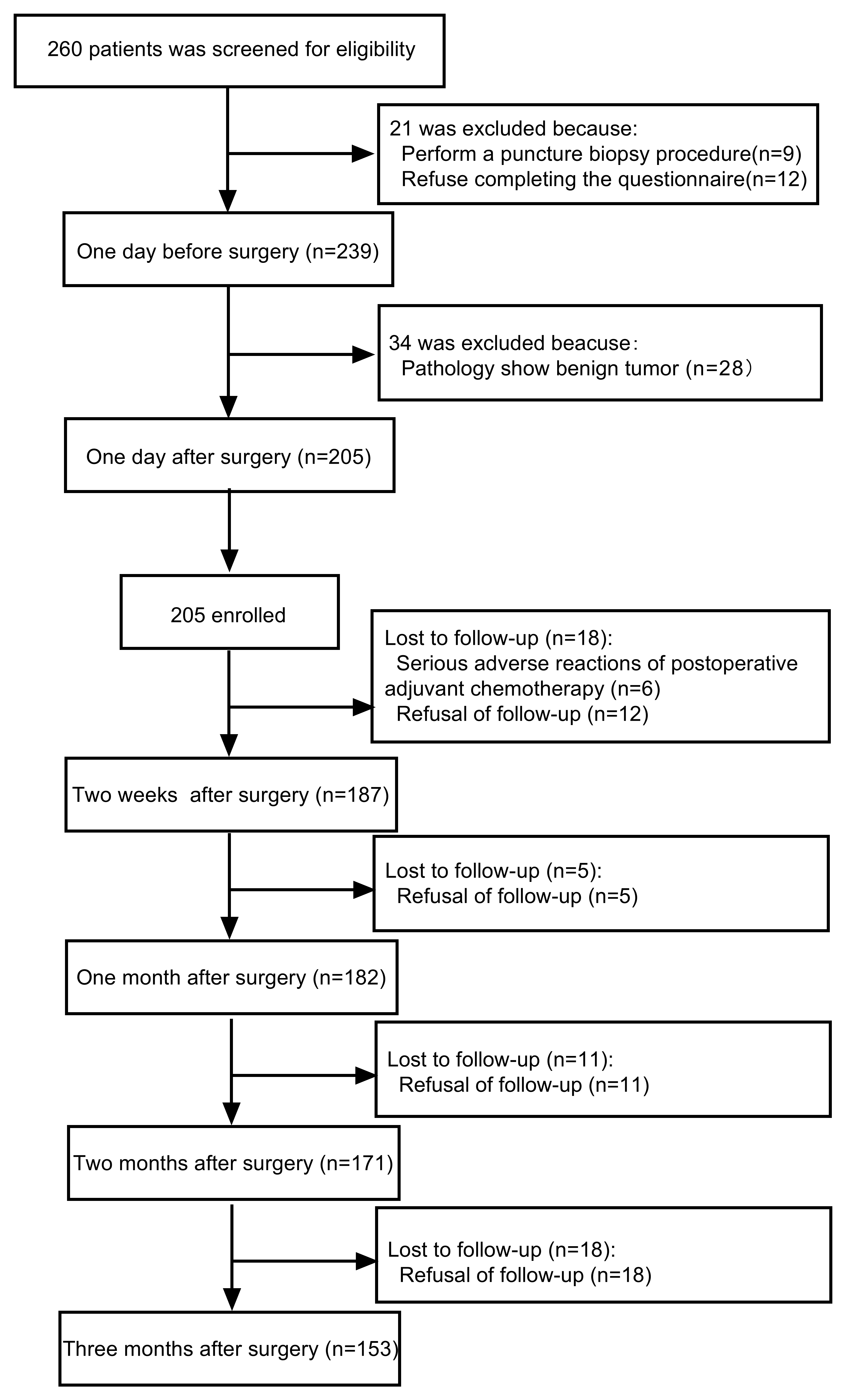

Supplement: Supplemental Material [file IANN_A_2479588_SM9967.zip › suppl_data/Supplementary_Figure_1.tif]
