# Supplementary material for: A score prediction model for predicting the heterogeneity symptom trajectories among lung cancer patients during perioperative period: a longitudinal observational study
Source: Ann Med. 2025 Mar 20;57(1):2479588. doi: 10.1080/07853890.2025.2479588 (PMC11934189; doi:10.1080/07853890.2025.2479588)
Supplement: Supplemental Material [file IANN_A_2479588_SM9967.zip › suppl_data/Clean copy - Supplementary_Table_1B.doc]

**Supplementary Table 1B** Incidence of severe symptoms in MDASI-TCM from preoperative to three months after surgery [n (%)].

| Symptom | T0 | T1 | T2 | T3 | T4 | T5 |
| --- | --- | --- | --- | --- | --- | --- |
| n(censored) | 205 (0) | 200 (5) | 187 (18) | 182 (23) | 171 (34) | 153 (52) |
| Pain | 0 (0.00) | 110 (55.0) | 27 (14.44) | 19 (10.44) | 7 (4.09) | 3 (1.96) |
| Fatigue | 0 (0.00) | 104 (52.00) | 39 (20.86) | 26 (14.29) | 10 (5.85) | 6 (3.92) |
| Nausea | 0 (0.00) | 19 (9.50) | 4 (2.14) | 2 (1.10) | 1 (0.58) | 0 (0.00) |
| Disturbed sleep | 0 (0.00) | 47 (23.50) | 23 (12.30) | 21 (11.54) | 13 (7.60) | 8 (5.23) |
| Distressed | 0 (0.00) | 19 (9.50) | 13 (6.95) | 7 (3.85) | 4 (2.33) | 3 (1.96) |
| Shortness of breath | 0 (0.00) | 28 (14.00) | 21 (11.23) | 19 (10.44) | 12 (7.02) | 5 (3.27) |
| Problem remembering | 0 (0.00) | 14 (7.00) | 9 (4.81) | 11 (6.04) | 10 (5.85) | 7 (4.58) |
| lack of appetite | 0 (0.00) | 25 (12.50) | 6 (3.21) | 7 (3.85) | 3 (1.75) | 2 (1.31) |
| Sleepy | 0 (0.00) | 36 (18.00) | 10 (5.35) | 6 (3.30) | 6 (3.51) | 3 (1.96) |
| Dry mouth | 0 (0.00) | 13 (6.50) | 12 (6.42) | 10 (5.49) | 6 (3.51) | 2 (1.31) |
| Sad | 0 (0.00) | 7 (3.50) | 7 (3.74) | 6 (3.30) | 4 (2.33) | 1 (0.65) |
| Vomiting | 0 (0.00) | 4 (2.00) | 3 (1.60) | 1 (0.55) | 4 (2.33) | 0 (0.00) |
| Numbness or tingling | 0 (0.00) | 4 (2.00) | 0 (0.00) | 2 (1.10) | 2 (1.17) | 1 (0.65) |
| Coughing | 0 (0.00) | 5 (2.50) | 18 (9.63) | 13 (7.14) | 10 (5.85) | 3 (1.96) |
| Expectoration | 0 (0.00) | 6 (3.00) | 14 (7.49) | 10 (5.49) | 9 (5.26) | 4 (2.61) |
| Palpitation | 0 (0.00) | 6 (3.00) | 4 (2.14) | 3 (1.65) | 2 (1.17) | 0 (0.00) |
| Problem with sweating | 0 (0.00) | 30 (15.00) | 6 (3.21) | 3 (1.65) | 6 (3.51) | 1 (0.65) |
| Problem with bitter taste | 0 (0.00) | 11 (5.50) | 7 (3.74) | 5 (2.75) | 5 (2.92) | 0 (0.00) |
| Mouth ulcer | 0 (0.00) | 1 (0.50) | 0 (0.00) | 2 (1.10) | 3 (1.75) | 0 (0.00) |
| Diarrhea | 0 (0.00) | 1 (0.50) | 0 (0.00) | 3 (1.65) | 2 (2.17) | 1 (0.65) |
| Constipation | 0 (0.00) | 8 (4.00) | 6 (3.21) | 4 (2.20) | 4 (2.33) | 0 (0.00) |
| Fidgety | 0 (0.00) | 4 (2.00) | 3 (1.60) | 3 (1.65) | 6 (3.51) | 0 (0.00) |
| Feverish sensation over  the palm and sole | 0 (0.00) | 8 (4.00) | 0 (0.00) | 4 (2.20) | 4 (2.33) | 0 (0.00) |
| Abdominal fullness | 0 (0.00) | 1 (0.50) | 3 (1.60) | 1 (0.55) | 2 (1.17) | 1 (0.65) |

MDASI-TCM,MD Anderson Symptom Inventory-Traditional Chinese Medicine.
