# Supplementary material for: A score prediction model for predicting the heterogeneity symptom trajectories among lung cancer patients during perioperative period: a longitudinal observational study
Source: Ann Med. 2025 Mar 20;57(1):2479588. doi: 10.1080/07853890.2025.2479588 (PMC11934189; doi:10.1080/07853890.2025.2479588)
Supplement: Supplemental Material [file IANN_A_2479588_SM9967.zip › suppl_data/Clean copy - Supplementary_Table_2.doc]

**Supplementary Table 2** Scores in MDASI-TCM from preoperative to three months after surgery（Mean±SD).

| Symptom | T0 | T1 | T2 | T3 | T4 | T5 |
| --- | --- | --- | --- | --- | --- | --- |
| n(censored) | 205 (0) | 200 (5) | 187 (18) | 182 (23) | 171 (34) | 153 (52) |
| Pain | 0±0.01 | 6.64±0.12 | 4.41±0.15 | 3.38±0.16 | 2.40±0.15 | 1.82±0.13 |
| Fatigue | 0.56±0.05 | 6.27±0.13 | 4.80±0.16 | 4.10±0.16 | 3.02±0.16 | 2.61±0.16 |
| Nausea | 0.66±0.07 | 4.94±0.16 | 3.64±0.18 | 3.05±0.19 | 2.43±0.19 | 2.04±0.19 |
| Disturbed sleep | 0.50±0.06 | 3.68±0.16 | 2.86±0.16 | 2.42±0.15 | 1.75±0.15 | 1.42±0.14 |
| Distressed | 0.18±0.03 | 4.30±0.15 | 4.12±0.16 | 3.66±0.16 | 3.02±0.16 | 2.61±0.16 |
| Shortness of breath | 0.61±0.08 | 2.51±0.17 | 2.28±0.17 | 2.03±0.16 | 1.95±0.17 | 1.68±0.16 |
| Problem remembering | 0.53±0.06 | 4.28±0.16 | 3.19±0.16 | 2.46±0.17 | 2.04±0.16 | 1.56±0.14 |
| lack of appetite | 0.10±0.03 | 2.37±0.13 | 3.38±0.16 | 3.23±0.16 | 2.35±0.16 | 1.74±0.14 |
| Sleepy | 0.12±0.03 | 2.32±0.13 | 2.76±0.16 | 2.42±0.16 | 1.68±0.16 | 1.03±0.14 |
| Dry mouth | 0.19±0.04 | 3.94±0.17 | 2.49±0.15 | 1.86±0.14 | 1.65±0.16 | 1.12±0.12 |
| Sad | 0±0.01 | 2.54±0.19 | 1.27±0.15 | 0.73±0.12 | 0.57±0.11 | 0.21±0.06 |
| Vomiting | 0.04±0.02 | 4.03±0.15 | 2.53±0.15 | 1.92±0.15 | 1.31±0.14 | 0.88±0.12 |
| Numbness or tingling | 0±0 | 3.94±0.17 | 2.65±0.17 | 1.95±0.15 | 1.51±0.16 | 0.98±0.13 |
| Coughing | 0.16±0.03 | 2.60±0.16 | 1.89±0.16 | 1.61±0.15 | 1.27±0.14 | 1.01±0.12 |
| Expectoration | 0±0 | 1.37±0.16 | 0.67±0.12 | 0.32±0.08 | 0.33±0.09 | 0.11±0.05 |
| Palpitation | 0.58±0.09 | 0.84±0.11 | 0.95±0.11 | 1.23±0.13 | 1.58±0.13 | 0±0 |
| Problem with sweating | 0.80±0.11 | 1.23±0.13 | 1.24±0.13 | 1.56±0.15 | 1.80±0.14 | 0.06±0.02 |
| Problem with bitter taste | 1.05±0.12 | 1.50±0.15 | 1.63±0.15 | 1.79±0.16 | 1.96±0.17 | 0.25±0.04 |
| Mouth ulcer | 0.14±0.04 | 0.36±0.09 | 0.31±0.09 | 0.27±0.07 | 0.22±0.07 | 0±0 |
| Diarrhea | 0.24±0.07 | 0.51±0.11 | 0.43±0.10 | 0.32±0.07 | 0.27±0.07 | 0±0 |
| Constipation | 0.88±0.11 | 1.29±0.14 | 1.49±0.13 | 1.82±0.15 | 2.35±0.14 | 0.31±0.05 |
| Fidgety | 0.93±0.13 | 1.29±0.15 | 1.37±0.14 | 1.61±0.15 | 1.91±0.14 | 0.12±0.03 |
| Feverish sensation over the palm and sole | 0.33±0.08 | 0.77±0.13 | 0.78±0.11 | 0.94±0.11 | 1.39±0.15 | 0.06±0.02 |
| Abdominal fullness | 0.42±0.08 | 0.78±0.11 | 0.81±0.11 | 1.02±0.12 | 0.79±0.10 | 0.02±0.01 |

MDASI-TCM,MD Anderson Symptom Inventory-Traditional Chinese Medicine.
