# Supplementary material for: A score prediction model for predicting the heterogeneity symptom trajectories among lung cancer patients during perioperative period: a longitudinal observational study
Source: Ann Med. 2025 Mar 20;57(1):2479588. doi: 10.1080/07853890.2025.2479588 (PMC11934189; doi:10.1080/07853890.2025.2479588)
Supplement: Supplemental Material [file IANN_A_2479588_SM9967.zip › suppl_data/Clean copy - Supplementary_Table_3.doc]

**Supplementary Table 3** Model Selection Criteria of the Growth Mixture Model (GMM) and Latent Class Growth Model (LCGM) analyses.

| Model | Parameter | Log(L) | AIC | BIC | aBIC | Entropy | LRT | LMR | BLRT | Class probability |
| --- | --- | --- | --- | --- | --- | --- | --- | --- | --- | --- |
| GMM |  |  |  |  |  |  |  |  |  |  |
| 2C | 19 | -3840.240 | 7718.479 | 7781.617 | 7721.418 | 0.869 | 0.240 | 0.240 | 0.000 | 0.14146/0.85854 |
| 3C | 23 | -3825.660 | 7697.320 | 7773.749 | 7700.878 | 0.874 | 0.397 | 0.379 | 0.000 | 0.07805/0.81951/0.10244 |
| 4C | 27 | -3813.220 | 7680.441 | 7770.162 | 7684.617 | 0.891 | 0.123 | 0.117 | 0.005 | 0.09268/0.76585/0.02927/0.11220 |
| 5C | 31 | -3806.016 | 7674.031 | 7777.044 | 7678.826 | 0.896 | 0.065 | 0.240 | 0.240 | 0.31220/0.15122/0.02927/0.05854/0.44878 |
| 6C | 35 | -3789.703 | 7649.406 | 7765.712 | 7654.820 | 0.926 | 0.585 | 0.574 | 0.000 | 0.03902/0.15122/0.32683/0.03415/0.02927/0.41951 |
| LCGM |  |  |  |  |  |  |  |  |  |  |
| **2C** | **13** | **-3974.644** | **7975.288** | **8018.487** | **7977.299** | **0.906** | **0.000** | **0.000** | **0.000** | **0.80488/0.19512** |
| 3C | 17 | -3894.896 | 7823.792 | 7880.283 | 7826.421 | 0.830 | 0.014 | 0.011 | 0.000 | 0.50244/0.14146/0.35610 |
| 4C | 21 | -3868.305 | 7778.609 | 7848.393 | 7781.857 | 0.782 | 0.153 | 0.143 | 0.000 | 0.20976/0.11707/0.41951/0.25366 |
| 5C | 25 | -3846.643 | 7743.286 | 7826.361 | 7747.153 | 0.812 | 0.206 | 0.193 | 0.000 | 0.21951/0.39512/0.03902/0.09756/0.24878 |
| 6C | 29 | -3837.457 | 7732.914 | 7829.281 | 7737.399 | 0.789 | 0.233 | 0.222 | 0.000 | 0.37073/0.07805/0.03902/0.08780/0.20976/0.21463 |

Note. AIC, Akaike Information Criteria. BIC, Bayesian Information Criterion. aBIC, sample-size adjusted BIC. LRT, likelihood ratio test. LMR, lo-mendell-rubin. BLRT, Bootstrapped-likelihood ratio test.1C, one-class. 2C, two-class. 3C, three-class. 4C,four-class. 5C, five-class. Log(L), .Log-Likelihood Function.
