# Supplementary material for: A score prediction model for predicting the heterogeneity symptom trajectories among lung cancer patients during perioperative period: a longitudinal observational study
Source: Ann Med. 2025 Mar 20;57(1):2479588. doi: 10.1080/07853890.2025.2479588 (PMC11934189; doi:10.1080/07853890.2025.2479588)
Supplement: Supplemental Material [file IANN_A_2479588_SM9967.zip › suppl_data/Supplementary_Table_1A.doc]

**Supplementary Table 1A** Incidence of symptoms in MDASI-TCM from preoperative to three months after surgery [n (%)].

| Symptom | T0 | T1 | T2 | T3 | T4 | T5 |
| --- | --- | --- | --- | --- | --- | --- |
| n(censored) | 205 (0) | 200 (5) | 187 (18) | 182 (23) | 171 (34) | 153 (52) |
| Pain | 1 (0.49) | 199 (99.50) | 178 (95.19) | 165 (90.66) | 140 (81.87) | 116 (75.82) |
| Fatigue | 89 (43.41) | 197 (98.50) | 180 (96.26) | 172 (94.51) | 149 (87.13) | 126 (82.35) |
| Nausea | 1 (0.49) | 120 (60.00) | 71 (37.97) | 38 (20.88) | 35 (20.47) | 16 (10.46) |
| Disturbed sleep | 79 (38.54) | 191 (95.50) | 157 (83.96) | 137 (75.27) | 114 (66.67) | 100 (65.36) |
| Distressed | 67 (32.68) | 173 (86.50) | 148 (79.14) | 133 (73.08) | 110 (64.33) | 90 (58.82) |
| Shortness of breath | 28 (13.66) | 186 (93.00) | 174 (93.05) | 167 (91.76) | 156 (91.23) | 130 (84.97) |
| Problem remembering | 64 (31.22) | 127 (63.50) | 118 (63.10) | 108 (59.34) | 103 (60.23) | 88 (57.52) |
| lack of appetite | 7 (3.41) | 181 (90.50) | 143 (76.47) | 112 (61.54) | 85 (49.71) | 55 (35.95) |
| Sleepy | 0 (0.00) | 166 (83.00) | 135 (72.19) | 112 (61.54) | 84 (49.12) | 57 (37.25) |
| Dry mouth | 77 (37.56) | 178 (89.00) | 155 (82.89) | 126 (69.23) | 113 (66.08) | 98 (64.05) |
| Sad | 24 (11.71) | 140 (70.00) | 104 (55.61) | 93 (51.10) | 78 (45.61) | 65 (42.48) |
| Vomiting | 0 (0.00) | 71 (35.50) | 36 (19.25) | 21 (11.54) | 19 (11.11) | 6 (3.92) |
| Numbness or tingling | 0 (0.00) | 104 (52.00) | 80 (42.78) | 68 (37.36) | 62 (36.26) | 48 (31.37) |
| Coughing | 13 (6.34) | 172 (86.00) | 172 (91.98) | 160 (87.91) | 139 (81.29) | 112 (73.20) |
| Expectoration | 21 (10.24) | 179 (89.50) | 157 (83.96) | 141 (77.47) | 104 (60.82) | 70 (45.75) |
| Palpitation | 10 (4.88) | 110 (55.00) | 89 (47.59) | 78 (42.86) | 80 (46.78) | 55 (35.95) |
| Problem with sweating | 29 (14.15) | 171 (85.50) | 134 (71.66) | 116 (63.74) | 96 (56.14) | 72 (47.06) |
| Problem with bitter taste | 34 (16.59) | 100 (50.00) | 89 (47.59) | 92 (50.55) | 89 (52.05) | 71 (46.41) |
| Mouth ulcer | 0 (0.00) | 14 (7.00) | 20 (10.70) | 18 (9.89) | 23 (13.45) | 12 (7.84) |
| Diarrhea | 0 (0.00) | 17 (8.50) | 23 (12.30) | 25 (13.74) | 30 (17.54) | 14 (9.15) |
| Constipation | 41 (20.00) | 139 (69.50) | 109 (58.29) | 96 (52.75) | 80 (46.78) | 59 (38.56) |
| Fidgety | 15 (7.32) | 115 (57.50) | 95 (50.80) | 85 (46.70) | 75 (43.86) | 56 (36.60) |
| Feverish sensation over  the palm and sole | 8 (3.90) | 79 (39.50) | 64 (34.22) | 52 (28.57) | 43 (25.15) | 24 (15.69) |
| Abdominal fullness | 4 (1.95) | 61 (30.50) | 74 (39.57) | 64 (35.16) | 54 (31.58) | 33 (21.57) |

MDASI-TCM,MD Anderson Symptom Inventory-Traditional Chinese Medicine.
