# Supplementary material for: A score prediction model for predicting the heterogeneity symptom trajectories among lung cancer patients during perioperative period: a longitudinal observational study
Source: Ann Med. 2025 Mar 20;57(1):2479588. doi: 10.1080/07853890.2025.2479588 (PMC11934189; doi:10.1080/07853890.2025.2479588)
Supplement: Supplemental Material [file IANN_A_2479588_SM9967.zip › suppl_data/Supplementary_Table_4.doc]

**Supplementary Table 4** Univariate logistic analysis of predictive factors for heterogeneous symptom trajectories among lung cancer patients during the perioperative period (n=205).

| Variables | | B | SE | X^2^ | *P* value | OR | 95%CI |
| --- | --- | --- | --- | --- | --- | --- | --- |
| Gender | |  |  |  |  |  |  |
|  | Female |  |  |  |  | 1.000 |  |
|  | Male | 0.256 | 0.356 | 0.516 | 0.472 | 1.291 | 0.643-2.593 |
| Age (years) | |  |  |  |  |  |  |
|  | ≤60 |  |  |  |  | 1.000 |  |
|  | ＞60 | 0.556 | 0.355 | 2.452 | 0.117 | 1.743 | 0.869-3.496 |
| BMI (kg/m^2^) | |  |  |  |  |  |  |
|  | ≥28 |  |  |  |  | 1.000 |  |
|  | 24.0-27.9 | -19.979 | 16408.711 | 0.000 | 0.999 | 0.000 | 0.000 |
|  | 18.5-23.9 | -0.178 | 0.580 | 0.094 | 0.759 | 0.837 | 0.268-2.610 |
|  | ＜18.5 | -19.979 | 16408.711 | 0.000 | 0.999 | 0.000 | 0.000 |
| History of lung disease | |  |  |  |  |  |  |
|  | No |  |  |  |  | 1.000 |  |
|  | Yes | 0.440 | 0.435 | 1.022 | 0.312 | 1.552 | 0.662-3.639 |
| History of hypertension | |  |  |  |  |  |  |
|  | No |  |  |  |  | 1.000 |  |
|  | Yes | -0.033 | 0.369 | 0.008 | 0.928 | 0.967 | 0.469-1.994 |
| History of diabetes | |  |  |  |  |  |  |
|  | No |  |  |  |  | 1.000 |  |
|  | Yes | 0.927 | 0.457 | 4.115 | 0.043 | 2.528 | 0.032-6.191 |
| History of smoking | |  |  |  |  |  |  |
|  | No |  |  |  |  | 1.000 |  |
|  | Yes | 0.337 | 0.359 | 0.879 | 0.349 | 1.400 | 0.693-2.832 |
| Personal history of malignant neoplasm | |  |  |  |  |  |  |
|  | No |  |  |  |  | 1.000 |  |
|  | Yes | -0.713 | 0.772 | 0.854 | 0.355 | 0.490 | 0.108-2.224 |
| Education status | |  |  |  |  |  |  |
|  | Middle school or below |  |  |  |  | 1.000 |  |
|  | High school or above | 0.231 | 0.353 | 0.430 | 0.512 | 1.260 | 0.631-2.517 |
| Occupation | |  |  |  |  |  |  |
|  | Office staff and worker |  |  |  |  | 1.000 |  |
|  | Farmer | 0.360 | 0.676 | 0.284 | 0.594 | 1.433 | 0.381-5.389 |
|  | Retirement | 0.212 | 0.417 | 0.258 | 0.612 | 1.236 | 0.546-2.798 |
|  | Self-employed people and others | 1.326 | 0.612 | 4.690 | 0.030 | 0.265 | 0.080-0.882 |
| Constitution | |  |  |  |  |  |  |
|  | Balanced constitution |  |  |  |  | 1.000 |  |
|  | Biased constitution | 1.055 | 0.508 | 4.314 | 0.038 | 2.872 | 1.061-7.771 |
| physical function | | 0.008 | 0.045 | 0.035 | 0.852 | 1.008 | 0.923-1.102 |
| role function | | -0.026 | 0.057 | 0.210 | 0.647 | 0.974 | 0.871-1.089 |
| social function | | -0.050 | 0.030 | 2.727 | 0.099 | 0.952 | 0.897-1.009 |
| cognitive function | | -0.015 | 0.018 | 0.750 | 0.387 | 0.985 | 0.951-1.020 |
| emotional function | | -0.017 | 0.015 | 1.251 | 0.263 | 0.983 | 0.955-1.013 |
| Extent of surgery | |  |  |  |  |  |  |
|  | Leaf cutting |  |  |  |  | 1.000 |  |
|  | Segmented cutting | -0.271 | 0.487 | 0.309 | 0.578 | 0.763 | 0.294-1.981 |
|  | Wedge-shaped cutting | 0.673 | 0.423 | 2.537 | 0.111 | 1.961 | 0.856-4.490 |
| Surgical duration time (h) | |  |  |  |  |  |  |
|  | ＜2 |  |  |  |  | 1.000 |  |
|  | ≥2 | 1.212 | 0.363 | 11.133 | 0.001 | 3.361 | 1.649-6.851 |
| Intraoperative blood loss (ml) | |  |  |  |  |  |  |
|  | ＜50 |  |  |  |  | 1.000 |  |
|  | ≥50 | 0.787 | 0.379 | 4.318 | 0.038 | 2.196 | 1.046-4.612 |
| Tumor location | |  |  |  |  |  |  |
|  | one lobe of left lung |  |  |  |  | 1.000 |  |
|  | one lobe of right lung | 0.347 | 0.379 | 0.839 | 0.360 | 1.414 | 0.674-2.970 |
|  | Bilateral lung lobes | 0.459 | 0.642 | 0.511 | 0.475 | 1.582 | 0.450-5.570 |
| Pathological type | |  |  |  |  |  |  |
|  | Adenocarcinoma *in situ* |  |  |  |  | 1.000 |  |
|  | Microinvasive adenocarcinoma | 0.675 | 0.551 | 1.501 | 0.220 | 1.964 | 0.667-5.784 |
|  | Invasive adenocarcinoma | 0.721 | 0.514 | 1.970 | 0.160 | 2.056 | 0.751-5.628 |
|  | Others | 1.034 | 0.647 | 2.558 | 0.110 | 2.812 | 0.792-9.987 |
| pTNM stage (8th edition) | |  |  |  |  |  |  |
|  | 0 |  |  |  |  | 1.000 |  |
|  | IA1 | -0.182 | 0.683 | 0.071 | 0.790 | 0.833 | 0.218-3.179 |
|  | IA2 | 0.866 | 0.539 | 2.586 | 0.108 | 2.378 | 0.827-6.835 |
|  | IA3 | 1.268 | 0.594 | 4.557 | 0.033 | 3.553 | 1.109-11.377 |
|  | Others | 0.916 | 0.596 | 2.361 | 0.124 | 2.500 | 0.777-8.044 |
| PLT (*10^9^/L) | |  |  |  |  |  |  |
|  | ＜190.5 |  |  |  |  |  |  |
|  | ≥190.5 | 0.956 | 0.559 | 2.924 | 0.087 | 2.602 | 0.870-7.783 |
| NEUT (*10^9^/L) | |  |  |  |  |  |  |
|  | ＜5.65 |  |  |  |  |  |  |
|  | ≥5.65 | 1.088 | 0.520 | 4.379 | 0.036 | 2.970 | 1.071-8.231 |
| WBC (*10^9^/L) | |  |  |  |  |  |  |
|  | ＜7.76 |  |  |  |  |  |  |
|  | ≥7.76 | 0.995 | 0.461 | 4.653 | 0.031 | 2.704 | 1.095-6.671 |
| Hb (g/L) | |  |  |  |  |  |  |
|  | ＜129.5 |  |  |  |  |  |  |
|  | ≥129.5 | 0.197 | 0.374 | 0.278 | 0.599 | 1.218 | 0.585-2.536 |
| TP (µmol/L) | |  |  |  |  |  |  |
|  | ＜69.85 |  |  |  |  |  |  |
|  | ≥69.85 | 0.689 | 0.387 | 3.160 | 0.075 | 1.991 | 0.932-4.255 |
| AST (U/L) | |  |  |  |  |  |  |
|  | ＜15.25 |  |  |  |  |  |  |
|  | ≥15.25 | -0.040 | 0.423 | 0.009 | 0.926 | 0.961 | 0.420-2.202 |
| Creatinine (µmol/L) | |  |  |  |  |  |  |
|  | ＜53.35 |  |  |  |  |  |  |
|  | ≥53.35 | 0.357 | 0.390 | 0.839 | 0.360 | 1.429 | 0.666-3.068 |
| LDH (U/L) | |  |  |  |  |  |  |
|  | ＜170.9 |  |  |  |  | 1.000 |  |
|  | ≥170.9 | 0.840 | 0.358 | 5.517 | 0.019 | 2.316 | 1.149-4.667 |
| Heparin binding protein (ng/L) | |  |  |  |  |  |  |
|  | ＜50.67 |  |  |  |  | 1.000 |  |
|  | ≥50.67 | 1.596 | 0.751 | 4.519 | 0.034 | 4.931 | 1.133-21.471 |
| FVC (%) | |  |  |  |  |  |  |
|  | ＜108.15 |  |  |  |  |  |  |
|  | ≥108.15 | 0.672 | 0.427 | 2.481 | 0.115 | 1.958 | 0.849-4.520 |
| FEV1 (%) | |  |  |  |  |  |  |
|  | ＜99.65 |  |  |  |  |  |  |
|  | ≥99.65 | 0.512 | 0.356 | 2.069 | 0.150 | 1.669 | 0.831-3.355 |

BMI, body mass index (calculated as weight in kilograms divided by the square of height in meters). pTNM, pathologic tumor-node-metastasis staging system.. PLT, platelet. NEUT, neutrophil. WBC, white blood cell. Hb, hemoglobin. TP, total protein. AST, aspartate aminotransferase. LDH, lactate dehydrogenase. FEV1, forced expiratory volume in the first second. FVC, forced vital capacity. OR, Odds Ratio.
